# Supplementary figures and images for: The draft genome of the microscopic Nemertoderma westbladi sheds light on the evolution of Acoelomorpha genomes
Source: Front Genet. 2023 Sep 26;14:1244493. doi: 10.3389/fgene.2023.1244493 (PMC10565955; doi:10.3389/fgene.2023.1244493)

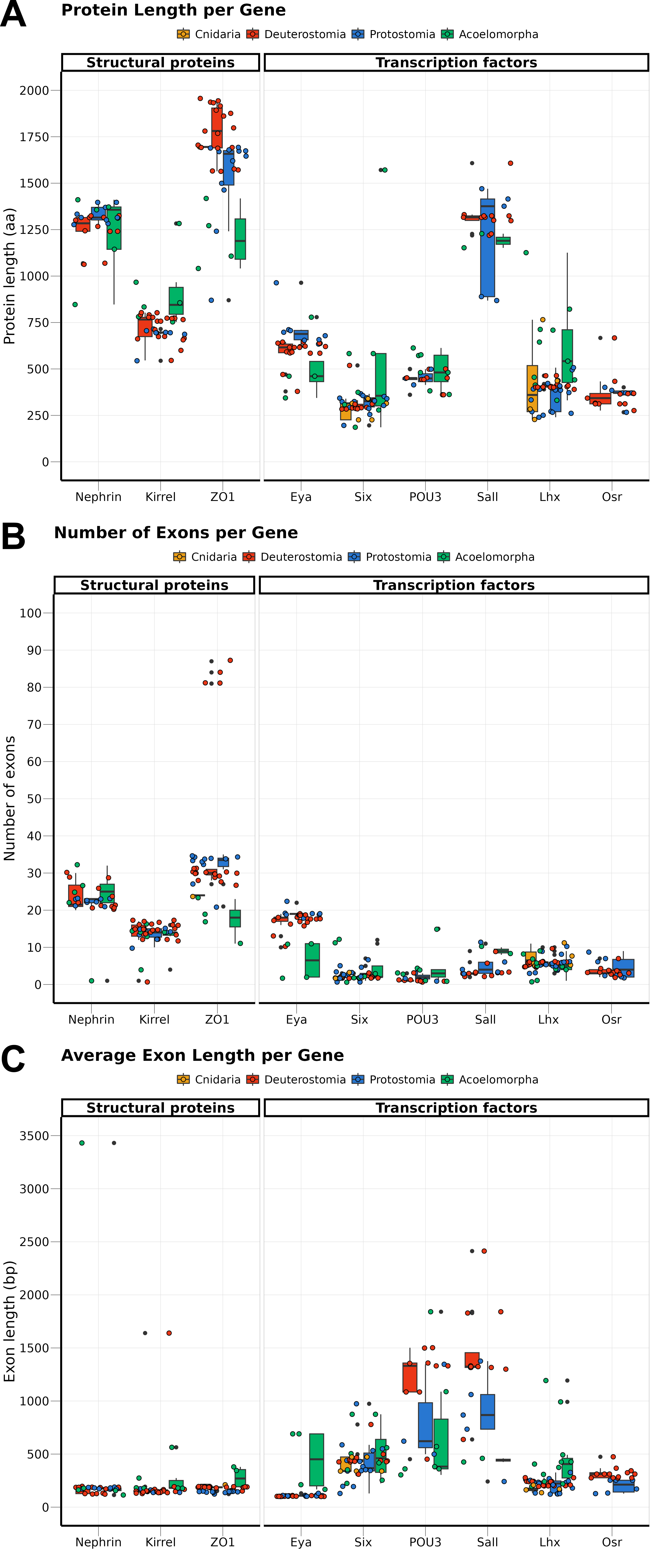

Supplement: Supplementary file 3 [file Image5.PNG]

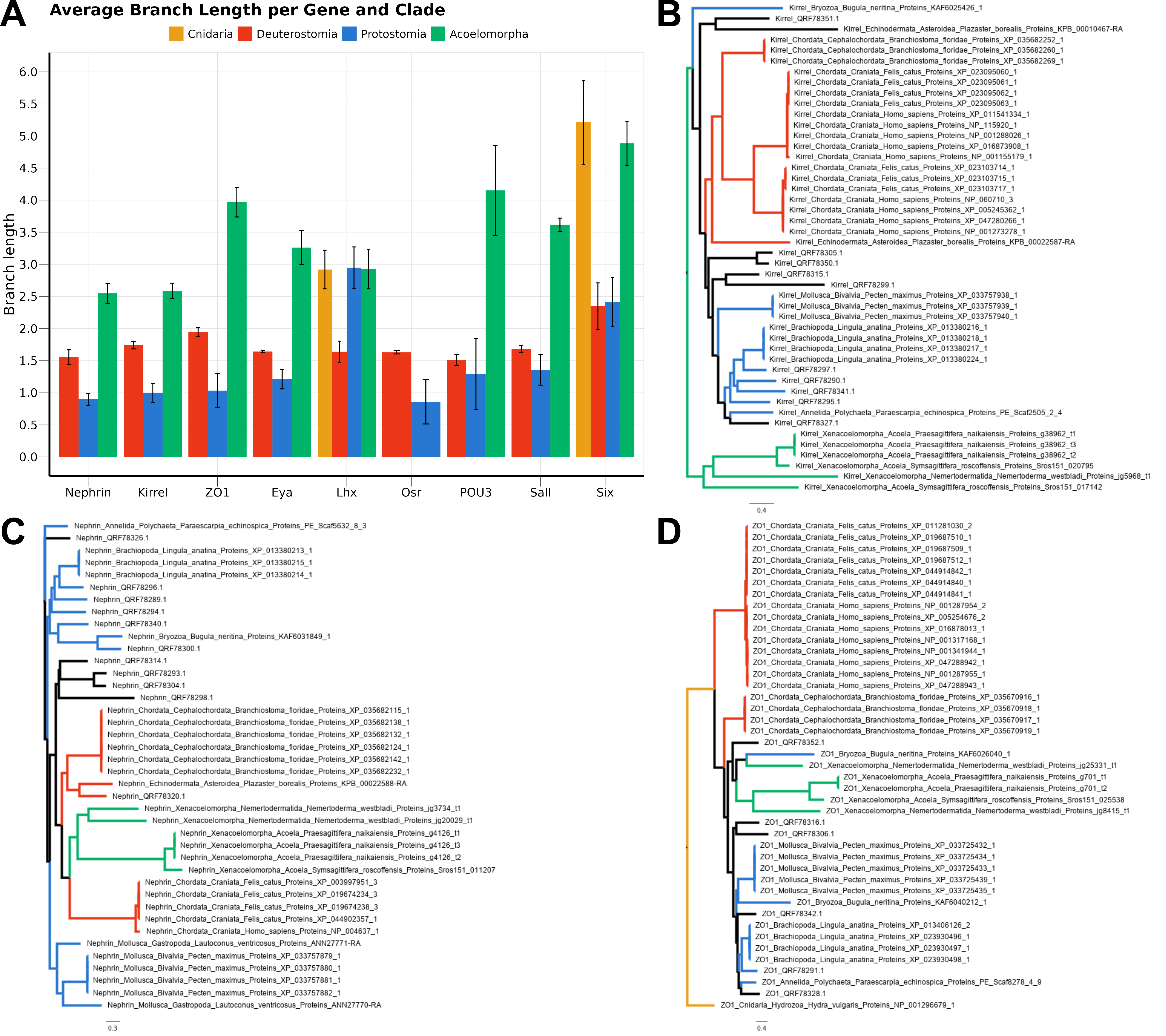

Supplement: Supplementary file 4 [file Image4.PNG]

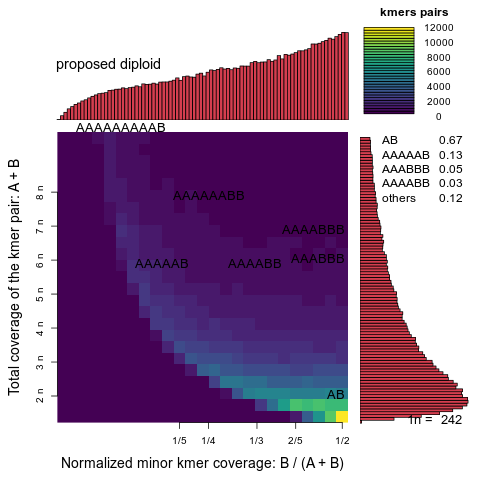

Supplement: Supplementary file 6 [file Image2.PNG]

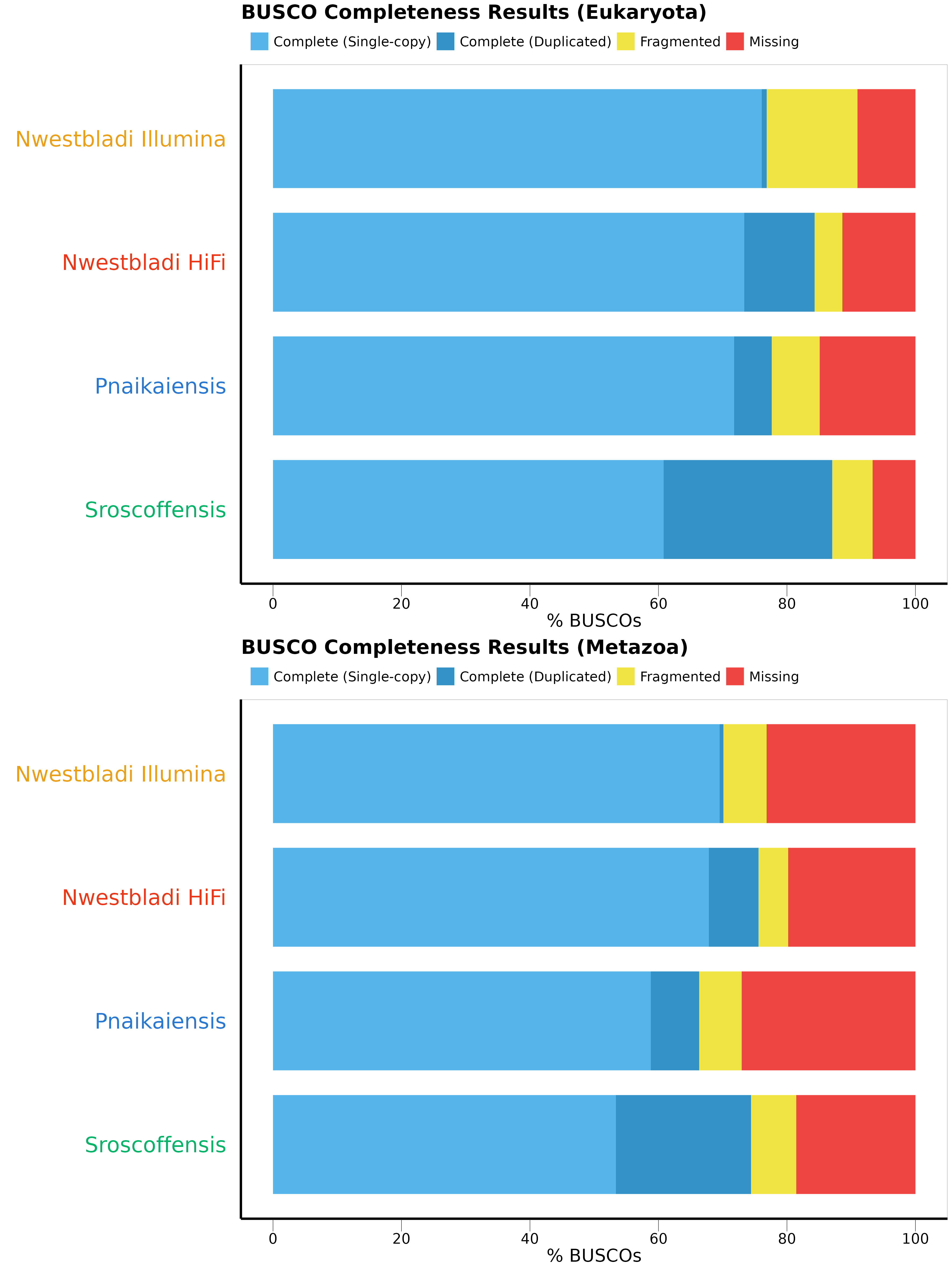

Supplement: Supplementary file 8 [file Image1.PNG]

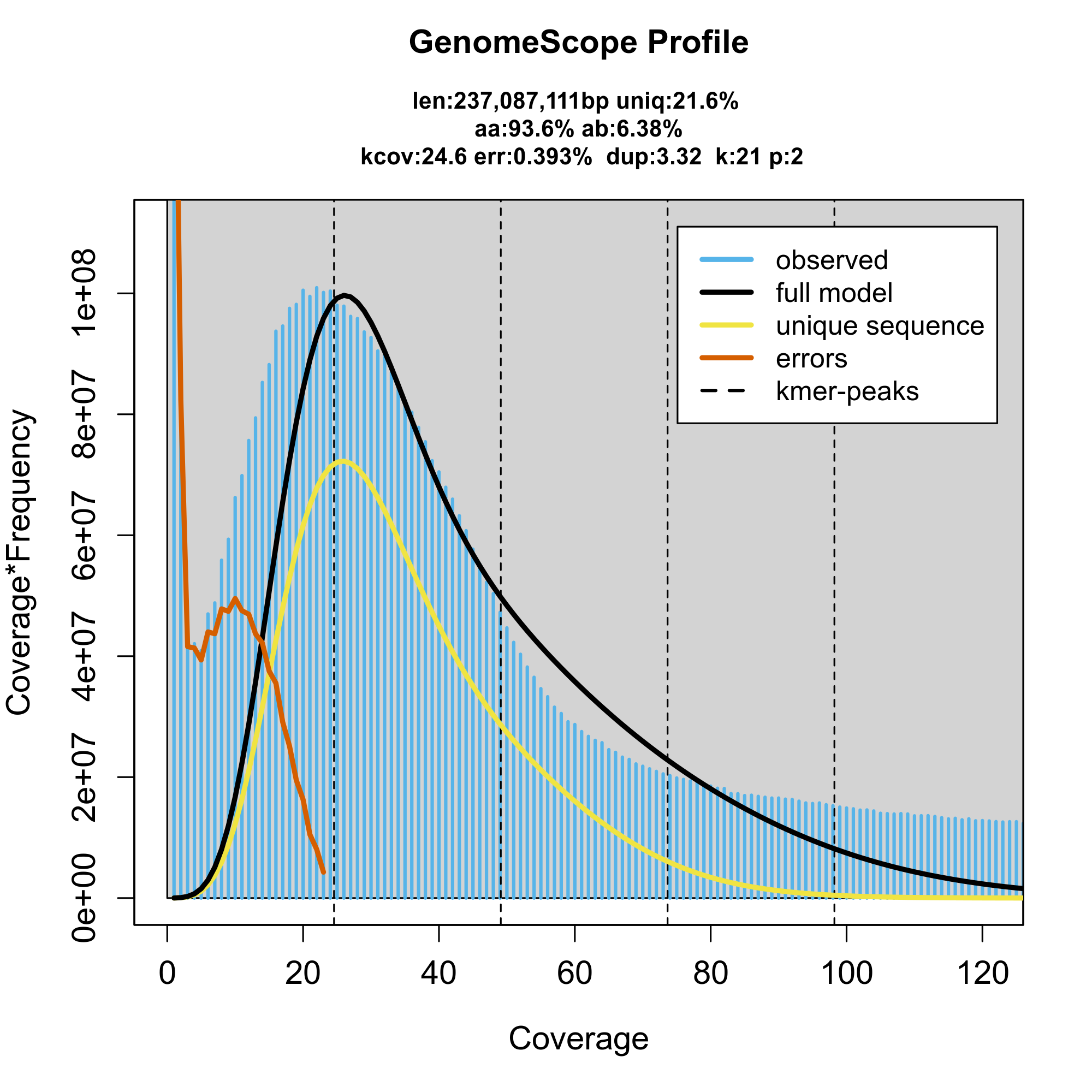

Supplement: Supplementary file 10 [file Image3.PNG]
